# 2454. Deep detection of antimicrobial resistance genes in hospital wastewater using the multiplex hybrid capture method

**DOI:** 10.1093/ofid/ofad500.2072

**Published:** 2023-11-27

**Authors:** Hiroaki Baba, Makoto Kuroda, Tsuyoshi Sekizuka, Hajime Kanamori

**Affiliations:** Tohoku University Graduate School of Medicine, Sendai, Miyagi, Japan; National Institute of Infectious Diseases, Shinjyuku, Tokyo, Japan; National Institute of Infectious Diseases, Shinjyuku, Tokyo, Japan; Tohoku University Graduate School of Medicine, Sendai, Miyagi, Japan

## Abstract

**Background:**

Wastewater can be useful in monitoring the spread of antimicrobial resistance (AMR) within a hospital. The abundance of antibiotic resistance genes (ARGs) in hospital effluent was assessed using metagenomic sequencing (mDNA-seq) and hybrid capture (xHYB).

**Methods:**

mDNA-seq analysis and subsequent xHYB targeted enrichment were conducted on two effluent samples per month from November 2018 to May 2021 from a sewer pipe connected to inpatient buildings at a university hospital with 1,200 beds and approximately 1,000 new admissions per month. Reads per kilobase per million (RPKM) values were calculated for all 1,272 ARGs in the constructed database. The monthly numbers of patients with presumed ESBL-producing and metallo-β-lactamase (MBL)-producing bacteria, methicillin-resistant Staphylococcus aureus (MRSA), and vancomycin-resistant enterococci (VRE) were compared with the monthly RPKM values of *bla*_CTX-M_, *bla*_IMP_, *mecA*, *vanA*, and *vanB* by xHYB.

**Results:**

The AMROTU hit count ratios for xHYB and mDNA-seq were well correlated (R2 = 0.99). The average RPKM value for all ARGs detected by xHYB was significantly higher than that of mDNA-seq (665,225 and 328, respectively, *p* < 0.05, Figure 1). The average RPKM values for *bla*_CTX-M_, *bla*_TEM_, *bla*_IMP_, *bla*_VIM_, *mcr*, *qnrS*, *aac(6’)-Ib*, *aph*, *ermB*, *ermF*, *tetM*, *sul1* and *sul2*, *mecA*, and *vanB* by xHYB were significantly higher than that of mDNA-seq (1,330, 9,120, 6,173, 224, 777, 1,272, 69,921, 61,927, 18,854, 1,885, 51,788, 6, and 125 vs. 1, 1, 4, 0, 0, 1, 30, 20, 16, 1, 22, 0, and 0, respectively, *p* < 0.05; Figure 1). The average number of patients with ESBL producers and RPKM values of *bla*_CTX-M-1_ genes for xHYB in 2020 were significantly higher than that in 2019 (17 vs. 13 patients per month and 921 vs. 232 per month, respectively, both *p* < 0.05, Figure 2). The average numbers of patients with MBL-producers, MRSA, and VRE were 1, 28, and 0 per month, respectively, while the average RPKM values of *bla*_IMP_, *mecA*, *vanA*, and *vanB* were 6,163, 6, 0, and 126 per month, respectively (Figure 2).

**Figure 1.**
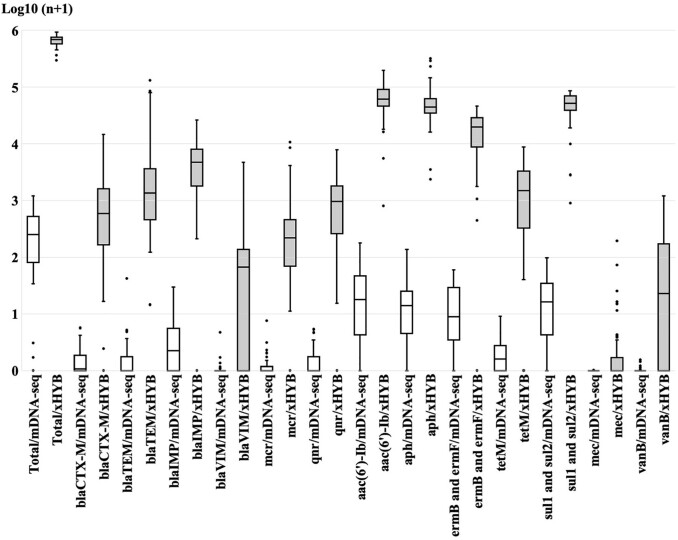
Comparison of reads per kilobase of gene per million (RPKM) value for each antimicrobial resistance gene (ARG) between metagenomic sequencing (mDNA-seq) and hybrid capture (xHYB).

In each box and whisker plot, the box marks the interquartile range; the horizontal line across the box shows the median. The white box indicates RPKM values of mDNA-seq and the gray box indicates that of xHYB.

**Figure 2.**
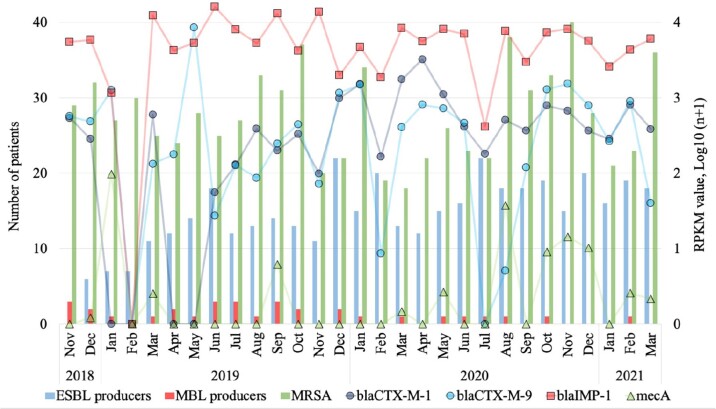
The monthly average number of patients with extended-spectrum β-lactamase (ESBL)-producing and metallo-β-lactamase (MBL)-producing bacteria, and MRSA, and the monthly Reads per kilobase of gene per million (RPKM) values of blaCTX-M-1, blaCTX-M-2, blaIMP-1 and mec A.

The left axis showed the number of patients, and the right showed the RPKM value, Log10(n+1). The blue, red, and green bars indicate the number of patients with ESBL and MBL-producers, and MRSA respectively. Purple and blue circle, red square, and the green triangle indicates RPKM values of blaCTX-M-1, blaCTX-M-2, blaIMP-1, and mecA, respectively.

**Conclusion:**

Monitoring ARGs in hospital effluent using xHYB was found to be more useful than conventional mDNA-seq in detecting ARGs including *bla*_CTX-M_, *bla*_IMP_, and *vanB*, which are important for infection control.

**Disclosures:**

**Hiroaki Baba, MD, PhD**, Amano Co., Ltd.: Grant/Research Support **Hajime Kanamori, MD, PhD, MPH**, Amano Co., Ltd.: Grant/Research Support

